# The anti-vaccination movement and resistance to allergen-immunotherapy: a guide for clinical allergists

**DOI:** 10.1186/1710-1492-6-26

**Published:** 2010-09-15

**Authors:** Jason Behrmann

**Affiliations:** 1 Programmes de bioéthique & Département de médecine sociale et préventive Faculté de médecine, Université de Montréal Pav. Margeurite d'Youville (7e étage) C.P. 6128, succursale centre-ville Montréal (Québec), H3C 3J7, Canada

## Abstract

Despite over a century of clinical use and a well-documented record of efficacy and safety, a growing minority in society questions the validity of vaccination and fear that this common public health intervention is the root-cause of severe health problems. This article questions whether growing public anti-vaccine sentiments might have the potential to spill-over into other therapies distinct from vaccination, namely allergen-immunotherapy. Allergen-immunotherapy shares certain medical vernacular with vaccination (e.g., allergy *shots*, allergy *vaccines*), and thus may become "guilty by association" due to these similarities. Indeed, this article demonstrates that anti-vaccine websites have begun unduly discrediting this allergy treatment regimen. Following an explanation of the anti-vaccine movement, the article aims to provide guidance on how clinicians can respond to patient fears towards allergen-immunotherapy in the clinical setting. This guide focuses on the provision of reliable information to patients in order to dispel misconceived associations between vaccination and allergen-immunotherapy, and the discussion of the risks and benefits of both therapies in order to assist patients in making autonomous decisions about their choice of allergy treatment.

## Review

Vaccination is the medical sacrament corresponding to baptism. Whether it is or is not more efficacious, I do not know.

Samuel Butler (1835-1902)

In 2009, the National Film Board of Canada and Play Films released the documentary film, *Shots in the Dark*[1], which showed interviews of parents of children that experienced severe cognitive and physical decline following immunization (better known as 'vaccination' amongst the lay-public and anti-vaccine proponents [2]). While the correlation between these harms and vaccination are purely anecdotal, the parents depicted in this documentary adamantly believe, due to their personal experience, that vaccines cause debilitating illness. Similar sentiments abound on the social networking website, *Facebook*®, where several hundred anti-vaccine fan-groups and discussion forums, with membership in the thousands, aim to inform the public of the dangers associated with this common public health intervention (search was performed by this author during December 2009, using the search term 'vaccination' with the *Facebook *search engine). In addition to social networks, internet searches using the term 'vaccination' with popular search engines now yield a majority of links to anti-vaccine websites [3]. These are but a few examples demonstrating a growing and highly visible anti-vaccine movement around the world [4], where the extreme and often unfounded fears and emotive discourse currently invoked in public debates concerning the safety of vaccines resemble mass-hysteria.

The consequence of growing resistance towards vaccination is the increase in morbidity and mortality from the resurgence of once uncommon infections, specific examples being recent epidemics of pertussis [5] and measles [6,7] in the developed world. This alone poses a formidable challenge to public health. It also worth questioning, however, whether challenges stemming from vaccine hysteria might be greater than initially thought: Can vaccine hysteria compromise health interventions *other *than vaccination initiatives? This article raises such a possibility by describing how the anti-vaccine movement may unexpectedly tarnish public perceptions towards allergen-immunotherapy, a treatment regimen for allergy which employs therapeutics that are *similar *to, yet *distinct *from, vaccines. Indeed, this article will demonstrate that propagandist anti-vaccination websites have started transposing vaccine-fears onto allergenic extracts and recommend that the public should refuse allergen-immunotherapy. Subsequent to descriptions of the similarities and differences between these therapeutic interventions, an overview of the anti-vaccine movement will provide a basis for an informational guide aimed at countering patient resistance to allergen-immunotherapy originating from the anti-vaccine movement.

Since the foundations of the anti-vaccine movement stem primarily from unfounded fears [4], many experts, but not all [8], recommend that health officials should focus on providing patients with reliable and truthful information about the risks and benefits of vaccination in order to counter current misconceptions [9,10]. The policy proposals herein concur with these recommendations, but are framed within the context of allergen-immunotherapy. Overall, this article aims to provide an informational guide for allergy specialists that can aid them in attending to patients' concerns about allergy treatment regimens that originate from vaccination-related fears, should clinicians encounter a vaccine-anxious patient in the clinical setting.

But first, the discussion will centre on identifying key similarities and differences between vaccination and allergen-immunotherapy.

## Vaccination and allergen-immunotherapy: When 'apples' seem like 'oranges'

### Vaccination

From the perspective of population health, the benefits accrued by humanity from the development and effective deployment of vaccination initiatives is immense and undeniable. One particularly reputable achievement has been the eradication of smallpox from the global population during the 1970's [11]. A multitude of once common vaccine-preventable diseases are following a similar path of diminution, such as measles, rubella and polio, which are now uncommon in the developed world [12,13] and increasingly less common in the developing world [14]. With a clinical history that dates over a century, a high vaccination rate of infants in the industrialized world, and an availability of annual vaccines against influenza, immunization efforts are by far the most well recognized public health intervention. However, vaccination initiatives have also been met with various degrees of public opposition throughout history, which will be described further below.

Vaccination--also described as 'shots', immunization, or inoculation--is a primary-level intervention that aims to prevent the initial emergence of disease. Preventing the transmission of infectious disease in this context resides in the controlled exposure of inactivated or weakened forms of infectious agents to the immune system, which in turn induces resistance (immunity). Early forms of vaccination involved invasive procedures that carried a significant risk for infection and produced permanent scars--the insertion of calf thymus particles into skin abrasions as a means for smallpox inoculation is but one example [15]. Current vaccination methods are benign in comparison, being typically administered by small injections. And certain inoculations are painless since they involve the ingestion of oral vaccines [16,17]. Since vaccines are solutions of labile biological material, they commonly contain preservative agents in order to retain their efficacy over time [18]. Other common vaccine additives are adjuvants, which are typically in the form of aluminium salts [19]. Adjuvants increase the reactivity (immunogenicity) of the vaccine by delaying the absorption of the active ingredients into the body, thus allowing for a prolonged interaction between the vaccine and the immune system. Therefore, adjuvanted vaccines typically require fewer injections (i.e., 'booster shots') in order to induce long-term immunity.

### Allergen-immunotherapy

Allergic sensitivities affect roughly 25% of the population of the developed world and cause numerous morbidities including hay-fever, skin rash, digestive disturbances, and allergy-induced asthma [20-23]. A variety of treatment strategies for allergy exist, which include pharmacotherapy, allergen avoidance and elimination, and allergen-immunotherapy (IT).

Similar to vaccination, IT has a lengthy clinical history that goes back nearly a century [24]. The therapy utilizes a class of therapeutics known as *allergenic extracts*, which are commonly referred to as 'allergy vaccines' or 'allergy shots' [25], pseudonyms that resemble terms often associated with therapeutics for vaccination. Indeed, allergenic extracts have a significant resemblance to vaccines and are administered by equivalent methods, primarily via injection but also increasingly by oral routes [26]. Furthermore, like vaccines, allergenic extracts often contain preservatives and adjuvants in order to increase their stability and therapeutic efficacy [27,28]. Yet, unlike injected vaccines, which are most often injected into muscle tissue or intra-dermally, allergen vaccines are administered subcutaneously. As their name implies, allergenic extracts are made by the extraction of allergens from biological sources (e.g., chemical extraction of cat allergens from cat hair clippings).

A typical IT regimen involves the gradual injection of increasing doses of allergens over the course of months and sometimes years. After multiple injections, physiological aspects of the immune system become altered and allergy-related IgE antibody levels are brought into balance with immune mediators that do not induce allergic responses and related histamine release (for a concise review, see: [29]). In other words, the therapeutic goal of IT is to induce an immune 'switch' or 'modification' away from allergic reactions as a means to induce tolerance. Therefore, in contrast to primary-level vaccination, IT is a tertiary-level health intervention, meaning that its goals are to diminish morbidities and negative health consequences of an illness already prevalent amongst the population. Relative to other allergy treatment strategies (e.g., pharmacotherapy, avoidance), IT has notable advantages. Of most significance is the fact that IT is the only treatment that can induce life long tolerance to (sometimes cure) allergic sensitivities, and thus can significantly reduce the need for consistent administration of costly drugs [30,31].

To this point, this author has focused on identifying key similarities and differences between vaccines and IT therapeutics (summarized in Table 1; note that the term "Allergen mixture" stated in the table refers to the biological components extracted from the biological source, which in turn contains both major and minor allergens--not to be confused with Mixed-versus Single-IT regimens). Many of these similarities could be readily identified by the lay-public, especially in terms of the names and administration routes used for both classes of therapeutics. However, IT and vaccination are radically different, especially in terms of the clinical/biomedical details of both therapies and the active ingredients used as therapeutics. It is unlikely that population groups other than clinicians and health officials would be fully cognizant of these important details. This then raises the reasonable possibility that the growing wave of public resentment and fear towards vaccination could 'spill-over' and influence public perceptions towards allergy treatments. A subsequent section of this article will demonstrate that the 'spill-over effect' has indeed begun. To conclude, vaccination is a proverbial 'apple' and IT is an 'orange'. While both share similarities in being 'fruits', they remain fundamentally different within a clinical context. Their similarities are, however, significant within a *population *context. Indeed, it is understandable that members of the lay-public are not adequately familiar with either therapy to be able to distinguish, say, 'vaccines' from 'allergen vaccines'. In the eyes of the public, apples likely appear equivalent to oranges and thus challenges originating from vaccine fears may well extend beyond that of vaccination.

**Table 1:** Similarities and differences between vaccination and allergen-immunotherapy

		Vaccination	Allergen-Immunotherapy
**Similarities**	*Clinical history*	Over a century	Nearly a century
	
	*Therapeutics contains adjuvants and preservatives?*	Yes, often	Yes, often
	
	*Synonyms: Medical and lay-public vernacular*	a) shots	a) allergy shots
	
		b) vaccines	b)allergen vaccines
	
		c) **IMMUN**ization	c) **IMMUN**otherapy
	
	*Administration*	Injection, occasional oral	Injection, occasional oral
	
	*Physiological target*	Immune system	Immune system

**Differences**	*Category of prevention*	Primary	Tertiary
	
	*Active ingredient*	Derivatives of infectious agent	Allergen mixture
	
	*Physiological response*	Induce immune response	Alter/modify immune response
	
	*Length of treatment*	Short, sometimes months	Lengthy, months to years
	
	*Number of injections*	Often single; may require 'boosters'	Multiple injections
	
	*Tissue injected*	Intra-muscular	Subcutaneous
	
	*Risk of anaphylaxis*	Extremely low	Low, but significant
	
	*Treatment goal*	Resistance/immunity to infection	Tolerance to allergen

### Side-effects from vaccination and immunotherapy: known, correlated, and unsubstantiated

As is the case for *all *categories of therapeutics, vaccines do occasionally cause side-effects and adverse drug reactions (ADRs) [32]. Most reactions are of little concern and remain localized at the injection site, such as pain, inflammation, and oedema. Within a minority of patients, certain vaccine recipients experience an allergic reaction that is often not due to the vaccine's active ingredients but rather its packaging, additives, or trace contaminants originating from the manufacturing process [18] (though for a minority of vaccines, the active ingredients can on rare occasions induce an allergic reaction, as is the case with tetanus and diphtheria toxoids [33]). For example, production of most influenza vaccines involves propagation of the virus within chicken eggs; some individuals have allergic sensitivities towards eggs and thus may develop a reaction to trace amounts of egg protein within the administered vaccine. *Severe *allergic reactions to vaccines do occur and can result in an anaphylactic reaction. Fortunately, anaphylactic and other severe reactions to vaccines occur at a rate of less than 1 per million administered doses [18], which signifies that mortality from vaccination is exceedingly rare [34]. To expand, estimates concerning the American population indicate that approximately 180 deaths from vaccination occur each year, which is roughly equivalent to the number traffic accident fatalities that occur every 1.5 days [35]. In addition to allergic reactions, possible vaccine contaminants have been correlated with a sudden rise in the incidence of a neurological condition known as Guillian-Barré syndrome (GBS) in America following the 1976 influenza vaccination campaign [36]. Subsequent flu vaccination campaigns have not been correlated with the syndrome [37,38]; thus, whether or not GBS is an ADR risk of vaccination remains debatable [39,40]. A final well-known--and ironic--vaccination risk concerns the possibility to transmit infectious disease from vaccines containing live active ingredients [18]. However, infections originating from live vaccines primarily occur in immuno-compromised and immuno-suppressed patients and thus, 'live' vaccines are contraindicated for this minority of the population.

Overall, the risks for serious ADRs to vaccines are arguably acceptable in terms of the population-level benefits that vaccination offers in preventing serious morbidity and mortality from infections, as well as providing the ability to "expand opportunities for health care by sparing ... resources that would otherwise be needed to care for individuals with preventable infectious diseases" [41] [p.487]. More importantly, relative to vaccines, rates of serious ADRs (e.g., death) are significantly higher for many widely prescribed medications [42] such as statins [43], blood thinners [44], antidepressants [45], but are routinely employed in clinical practice despite these known risks. To conclude, the relatively low risks of complications associated with vaccination are arguably acceptable and should not discourage their use in the general population.

Additional pathologies pertaining to severe cognitive and physical disability have been observed to coincide temporally with the administration of vaccines. However, the suggested correlations between these medical anomalies and vaccination are unsubstantiated and, at best, purely anecdotal [18,46,47]. One notable, but thoroughly debunked, example pertains to autism in children, where the mercury-containing vaccine preservative, *thimerosal*, was one of many [46] purported vaccine-related risk factors in the development of this disorder. Others have suggested that the multitude of vaccines used in childhood immunization programs are too numerous and thus might 'overload' a child's developing immune system. One suggested result of this overload might be an increased risk for immune disorders such as allergy and allergy-induced asthma. Additional examples include correlations with diabetes, multiple sclerosis, and sudden infant death syndrome. The tentative associations between vaccination and these pathologies have since undergone extensive evaluation through a variety of methods at independent research institutes. The results from these studies discredit the association of these illnesses with vaccination [18,46-48]. It is also important to note that amidst much media frenzy, the initial research article that suggested a link between vaccination and autism was retracted from *The Lancet *for numerous reasons ranging from unethical research practices, conflicts of interest undeclared by the authors, and questionable scientific methodology [49-51]. (Note that the lead author at the centre of this controversy, Dr. Wakefield, recently lost his license to practice medicine in the United Kingdom [52]).

There are several notable ADRs associated with allergen vaccines used in IT as well. The majority of adverse reactions observed are similar to those described previously for vaccines, being pain, inflammation, and oedema localized at the site of injection [53]. However, during the initial phase of therapy these reactions are often in greater magnitude than those observed with regular vaccines, which is understandable since IT functions through the injection of allergenic therapeutics into an allergen-sensitized patient. For adults, these localized adverse reactions are simply unpleasant, yet can be a cause for significant psychological stress when experienced by children [54]. It is important to note that allergic reactions to IT therapeutics are: 1) expected, 2) originate from the active ingredients of the therapeutic, 3) and are an unavoidable aspect of the therapy. This is in sharp contrast to allergic reactions to vaccines, which are unexpected, uncommon, and primarily due to additives or trace contaminants in the final therapeutic. As is the case with vaccines, life-threatening allergic reactions such as anaphylaxis can occur during the course of IT. However, since IT necessitates multiple injections of an allergenic compound, the incidence of anaphylactic reactions is far greater than that observed with vaccines, i.e., estimated to range between 6 events for every 100 injections [55] to 6 events for every 1000 injections [56]. These risks are well known, and clinicians providing IT are strongly encouraged to follow strict practice guidelines that minimize adverse reactions to IT [29,57,58]. When administered safely, deaths from IT are extremely rare. Unlike vaccines, there are no reports of severe cognitive or physical disabilities associated with the administration of IT therapeutics. However, two case reports representing two patients, one for scleroderma [59], the other for Sjögren's syndrome [60], associated temporally the onset of these diseases with IT, though exact causation was not established.

Building from the previous description of the risks of ADRs with vaccination, the discussion will now focus on the growing public sentiments against vaccination.

## The foundations of the anti-vaccine movement

Waves of public resentment and fears centering on vaccination are not a modern phenomenon, but rather one that has reappeared throughout the history of this intervention [61]. Unlike the earlier vaccination efforts against smallpox during the 1800's, where anti-vaccine propaganda was disseminated via posters and newspapers, proponents against vaccination now have numerous additional means to communicate their positions to the general public, the Internet being of particular importance [3,4,62,63]. It is important to note that the growing plethora of anti-vaccine websites exist at a time where millions of people are using the Internet as a means to obtain medical information [64].

Studies that analyzed the content of anti-vaccine websites indicate that anti-vaccine proponents vocalize a minority of justifiable criticisms alongside a majority of manipulative information [3,4,8,62,63]. For example, many criticisms stem from ethical issues in relation to imposed vaccination and the loss of civil liberties, as well as avoiding unnecessary vaccine-risks in the absence of infection. Indeed, coercive vaccination policies do exist, such as restrictions in school enrolment for unvaccinated children [65], and many people view these policies as unethical. However, vaccine opponents equate most vaccination programs with severe forms of government oppression and often omit the fact that most vaccination programs involve voluntary compliance; only rarely is vaccination obligatory. Moreover, purported claims that vaccines are currently unnecessary are uncorroborated. Indeed, certain vaccine-preventable diseases are not overtly prevalent, but this does not mean that they no longer exist within society. Vaccine opponents also commonly note undisputed vaccine-ADRs, including allergic reactions, infections, and death. However, these anti-vaccine websites grossly exaggerate the incidence of such rare ADRs.

Propagandist information is another commonality shared by anti-vaccine websites [3,4,8,62,63]. While discredited by reliable scientific evidence, vaccine-opponents remain adamant that inoculation is the cause of debilitating diseases such as autism and multiple sclerosis. Others still claim that multiple vaccines can 'overload' the immune system and is the cause of allergy, and in general, vaccination is 'fundamentally unnatural'. Many sites report very emotional stories of vibrant, healthy children that succumbed to horrific illnesses or death following the administration of common childhood vaccines, but they do not demonstrate a causative link between the two events. Finally, many make claims that vaccination efforts are fraught with controversy and describe elaborate conspiracy theories that explain the 'true' motives underlying vaccination policies. Popular conspiracy theories include: assertions that vaccines are ineffective and that infections began to disappear prior to vaccination; governments and scientists are hiding evidence of the actual harms caused by vaccines; vaccine efforts are schemes to generate profits for large pharmaceutical companies; and that vaccine initiatives are means to conduct genocide.

It is unknown to what *extent *anti-vaccine propaganda disseminated through media outlets or the Internet is undermining public trust in vaccination. Numerous surveys suggest that it is significant. At a minimum, anti-vaccination websites are observed to influence public perceptions towards vaccination, where parents whom exempt their children from receiving common vaccines often have obtained information from such Internet sources [66]. Furthermore, one study [67] demonstrated that up to half of American survey respondents refused the annual influenza vaccine due to the belief that they would develop influenza disease from the vaccine. Another American study [68] found that 15% of parents of young children did not want their child to receive any of the recommended childhood inoculations. Moreover, it is incorrect to assume that anti-vaccine sentiment is isolated amongst uneducated people or certain minority groups that share radical ideologies. Rather, a significant proportion of American supporters of the current anti-vaccination movement are of members of the middle class and have some level of university education [69]. By and large, these studies suggest that anti-vaccine sentiment exists throughout society, where the unfounded fears and anxiety now associated with vaccination could constitute a form of mass-hysteria. When taken as a whole, the arguably irrational nature of vaccine hysteria should raise concerns about whether other 'vaccine-like' medical interventions may also become tarnished in the public eye, as is argued here concerning IT. Indeed, information found by this author on the Internet indicates that public vaccine-fears and vaccine-opposition have started being transposed onto IT and allergy therapeutic regimens.

Replicating website searches conducted by Kata [8] and Wolfe and colleagues [2,63], and using search terms such as "anti-vaccination, vaccine, allergy, immunotherapy" in March 2010, yielded anti-vaccine websites and Internet blogs that have begun discussions questioning the safety and utility of IT. (A detailed quantification of these websites is beyond the scope of this article, but would be an interesting topic for future investigations). Many sites also confuse vaccination ADRs with IT treatments and purport manipulative and/or false information concerning IT and allergies. One notable example is blog entries [70] from the site, http://m.digitaljournal.com. What appears to be a blog entry from a member of the general public whose child received IT demonstrates that vaccine ADRs and related fears are being mistakenly associated with allergenic extracts--this entry relates to bacterial contamination of vaccines and the possible link with Guillain-Barré Syndrome (GBS):

"... after reading this report and reading there might have been bacterial contaminant in the H1N1 vaccine makes me wonder if there could have been bacterial contaminant in the allergy shots."

A subsequent entry on the same blog employs scientific jargon and claims that allergenic extracts contain the notorious "autism-causing" preservative, thimerosal:

*"... if your son received an allergy shot from a multi dose vial, he*(sic) *more than likely had thimerosal in it. By weight thimerosal is 40.7% mercury. Mercury is a neurotoxin and can affect many areas of your body."*

Another blog entry [71] from the website, http://childhealthsafety.wordpress.com, demonstrates similar convoluted and mistaken associations between vaccines and allergenic extracts (skin prick tests are clinical assays using allergenic extracts [e.g., peanut extract] in order to diagnose allergen sensitivities [e.g., peanut allergy]; the underline emphasis was added by this author):

"Vaccines are the direct cause of the food allergy epidemic. Why are the manufacturers of vaccines allowed trade secret protection for vaccine ingredients? Why is peanut oil considered safe to inject along with aluminum based on studies where children eat the oil or based on the skin prick test? IT ISN'T THE SAME!! The fatal food allergies are directly caused by vaccines!! The evidence is there."

Certain websites of supposed specialists in complementary and alternative medicine encourage patients to reject IT in favour of treatments such as homeopathy and often purport mistaken facts about IT and vaccination. Entries [72] within the website, http://e-holistichealth.blogspot.com, are exemplary (underline emphasis added):

(This entry compares allergenic extracts to vaccines) *"Allergy shots are often called "vaccines" because (1) they are injected and (2) the intention of both is to confer immunity."*

"... allergy shots must stop after 3 to 5 years and at that time the doctor has to decide whether to continue them or not. That would suggest that the cumulative effect of getting allergy shots compromises immune function in some way or has other side effects."

"Both allergy shots and vaccines have risks for allergic reactions, including anaphylaxis. The risk is higher and more common with vaccines (for obvious reasons)."

"...[IT] *therapy only lessens the severity of the allergy response and creates other side effects (headaches, skin conditions, additional allergies)."*

"Neither vaccination or allergy immunotherapy addresses the underlying organ weaknesses and immune system problems that make the person susceptible to infections and allergic reactions."

As a final example, the popular and notorious anti-vaccination website, Vaccination Liberation (http://www.vaclib.org), warns the public to reject allergy-vaccines and that the common aluminum salt adjuvants in allergenic extracts are of significant toxicological concern [73] (for an analysis of the website, Vaccination Liberation, see: [8]). Overall, this overview of Internet-based information indicates that mistaken associations between IT, vaccine-fears and the anti-vaccination movement are a current reality.

## Countering patient fears: a practical guide for clinical allergists

The final section of this article will now outline an informational guide to counter possible patient distrust of IT originating from the anti-vaccine movement. Policy recommendations aimed at addressing public fears towards vaccines have been proposed in the medical literature [9,10]. In brief, these recommendations emphasize that patients are in need of reliable, understandable and trustworthy information concerning immunization in order to dispel common misconceptions associated with the intervention. Such a strategy is also pertinent in relation to anti-vaccine sentiments that unduly tarnish IT; information is key. Indeed, clinicians should be prepared to suggest to patients where they can find reliable information on the Internet (for example, by referring patients to the websites of the Canadian Society of Allergy and Clinical Immunology [74], or the American Academy of Allergy, Asthma and Immunology [75]). Yet, while the Internet is a widely used public source of medical information, it is invariably the starting point for - and not a replacement of - seeking advice from a trusted health professional.

Clinicians providing IT should be informed of the effect vaccine fears may have on their clinical practice. For one, clinicians specializing in allergy treatments may be caught off-guard when encountering a patient that is fearful of allergy therapeutics because of vaccine anxiety. Clinicians may not be able to immediately understand the underlying connections or reasons for these fears, especially since an allergist knows that vaccination and IT are fundamentally different therapies. Furthermore, allergy specialists may not be adequately familiar with the details of vaccination and the growing anti-vaccine movement, which is understandable since vaccination is typically not directly related to the treatment of allergic sensitivities; more generally, it is the case that many health care workers are unfamiliar with details concerning vaccination and vaccine safety [4].

What information, then, is necessary and how should it be conveyed to patients? The following section provides an informational guide for clinicians, structured in the form of hypothetical questions vocalized in lay-public language that address basic fears and misconceptions concerning vaccination. Suggested methods to address these questions are derived from the information provided in the previous sections of this article.

### 1) Shots, allergy shots: What's the difference?

Clinical allergists will likely face a particular challenge in communicating a simple explanation as to why immunization/vaccination/vaccines/shots are fundamentally different from immunotherapy/allergy-vaccines/allergy-shots. It is thus of utmost importance that allergy specialists are informed about the details of vaccination and any associated fears. This should include familiarization with common vaccine additives and adverse drug reactions. Only then will clinicians have the trustworthy and reliable information needed to provide a detailed comparison between each therapy, and so not be caught off-guard by questions related to vaccine fears. Allergy specialists should be prepared to use their clinical knowledge of IT to demonstrate the *absolute *differences between vaccines and allergen vaccines. Recall that the main *differences *between vaccination and IT are evident within a clinical context that will unlikely be common knowledge to members of the general public (see Table 1). Clinicians should thus focus on describing these 'non-obvious' clinical details in a readily understandable manner. For example, patient-oriented discussions could describe the difference between 'allergen-tolerance' versus 'immunity', and explain that allergen vaccines only contain allergens; there is thus *no *risk of transmitting infection with these drugs though this small risk does exist with certain live vaccines. Of course, in an effort to provide truthful and balanced information, clinicians should not down-play any of the *similarities *between vaccines and allergen-vaccines (e.g., both contain adjuvants and preservatives), as well as not hesitate to state that the risk of adverse reactions associated with IT is greater than that of vaccination (though both have excellent records of safety and efficacy, especially in terms of vaccination).

### 2) Do allergy vaccines contain harmful additives?

This concern stems from real (e.g., allergic reaction to additives) and unfounded (e.g., thimerosal, mercury, and autism) risks related to vaccine ingredients. Clinicians need to be informed of details of vaccine additives and should be able to compare these with common additives used in IT therapeutics. For example, allergy specialists should be prepared to respond to basic questions concerning thimerosal and mercury (e.g., vaccine manufacturers have voluntarily stopped using thimerosal in most vaccine formulations [18,46]). Another example is that clinicians should offer relevant comparisons such as: allergen vaccines do not contain mercury metal but often have harmless aluminium salts as adjuvants. Lastly, clinicians should know if additive-free versions of allergy vaccines are available in case a patient is adamantly opposed to particular additives.

Of additional importance, clinicians should be able to provide a basic level of information that will dispel common misconceptions linking vaccine additives and serious illness, as well as noting the true frequency at which side effects, like allergic reactions, occur. However, vaccine-risks are not equivalent to allergen-vaccine-risks and this should be clearly explained. For example, vaccine-related allergic reactions are unexpected, uncommon, and most often due to additives or trace contaminants; IT-related allergic responses are expected, caused by the active ingredients, are an unavoidable aspect of the therapy, and treatments are medically supervised in order to minimize the risk of serious harm.

### 3) Is this therapy unnecessary and a method for pharmaceutical companies to make money?

This question represents one of many popular conspiracy theories purported by vaccine opponents. In general, the efficacy and utility of vaccines are claimed to be false and correspondingly, there are ulterior motives underlying the administration of vaccines, which in this case relates to profiteering. Thus, allergy specialists should be prepared for outlandish conspiracies and not simply 'laugh-off' these irrational theories, but rather counter them with rational arguments. In relation to the above example, clinicians should note that IT aims to induce long-term tolerance and can reduce the need for consistent administration of costly allergy drugs that only transiently reduce symptoms (for instance, a recent study [31] demonstrated that immunotherapy-treated patients had significantly lower 18-month median per-patient total health care costs ($3,247 versus $4,872)). This medical goal runs counter to efforts to generate profits through consistent drug consumption. The same argument applies to vaccination, being a cost-effective means to reduce health care expenditures that would otherwise be needed to treat infectious disease.

### 4) Will this treatment 'overload' my immune system?

Common criticisms of vaccination are that it is unnatural, and multiple vaccinations in particular are claimed to produce immune dysfunction. The unfounded concern that multiple vaccinations can 'overload' the immune system is particularly pertinent to IT. Unlike vaccination, which typically requires one or few injections, IT necessitates several injections over the course of months or years. The appearance of overloading the body with allergen-vaccines will likely seem even more pronounced with this treatment relative to common vaccination programs; this issue merits particular attention. Clinicians should thus be prepared for patient concerns of 'overloading the immune system' and be able to respond to such fears. One strategy to attend to this concern is for a clinician to rehearse means to communicate with the patient as to why multiple injections are needed as a means to induce tolerance. Certain IT treatments require fewer injections, like rush-immunotherapy [58], and clinicians should be prepared to recommend these alternatives to patients fearing multiple injections (if the therapy is available). Lastly, clinicians should be prepared to respond to these concerns with rational arguments, such as by informing the patient that our immune systems are bombarded daily with numerous, naturally occurring pathogens (moulds, bacteria, viruses). These daily immune responses do not 'overload' one's immune system, therefore why should the occasional IT injection do so?

### 5) Will there be consequences if I refuse or stop treatment (i.e., restrictions in school enrolment)?

This fear focuses on coercive or mandated vaccination policies and a perceived attack on civil liberties. The negative sentiments stemming from the perception of being forced to undergo an unwanted medical intervention is the source of much anti-vaccination rhetoric. Clinicians need to be aware of how patients may mistakenly think they are being forced or coerced into treatment and be ready to assert that patients are free to stop treatment whenever they choose. Clinicians should inform patients that their treatment will remain confidential and that third parties, such as government officials, will never know whether or not they received treatment. It might also prove helpful to inform patients fearful of coercion that their allergy poses no direct harm to others, and thus, there is no need for third parties to impose treatment under any circumstance.

### 6) Will I have an allergic reaction or develop additional allergies from this treatment? Will I have a bad reaction to the therapy? Can it kill me?

These questions exemplify how certain fears towards vaccination can be partly justified as well as partly unfounded, and share a common theme. Overall, anxieties concerning adverse drug reactions, such as severe allergic reactions and death, are partly do to the *over*-statement of actual vaccination risks by anti-vaccine proponents. Additionally, clinicians will likely be caught off-guard by a patient's assumption that an allergy treatment might give them *more *allergies. Therefore, clinicians should be prepared to explain how these assumptions stem from unfounded fears that vaccines cause immune disorders and be prepared to assert that a properly conducted IT regimen is a treatment that will not result in additional allergies.

Fears of severe reactions and death stemming from vaccination are particularly important in relation to IT because the well-known and severe ADRs for both therapies are roughly equivalent (e.g., mortality risks for both therapies are primarily due to anaphylactic reactions). Therefore, clinicians should be prepared to explain that risk of death from anaphylaxis is indeed a well-known concern, but is still very rare for both IT and vaccination. Second, it is noteworthy that allergic reactions in IT, unlike vaccination, are a recognized (and planned for) unavoidable aspect of therapy and these reactions are typically not severe; the patient should be made aware of this fact. If the vaccine-anxious patient cannot be convinced that minor risks of ADRs with IT are arguably acceptable, the clinician should support the patient in choosing alternate therapies (i.e., pharmacotherapy). Third, when encountering a vaccine-anxious patient, clinicians should provide an at-length discussion concerning the detailed practice protocols that are followed in IT and that these protocols (e.g., supervision following therapy), strongly recommended by the allergology community as imperative, are indeed effective in significantly reducing the risk of serious complications and death. (Regardless, this discussion is necessary to enable the informed consent of the patient in the first place.) It is important that clinicians are aware of the fact that the risk of anaphylaxis is higher for IT than vaccination and to not hide this fact from patients raising concerns towards vaccines. Overall, clinicians should know not to trivialize or omit discussion of any risks with IT, no matter how minor, since vaccine opponents have mislead many people into believing that minor risks are major concerns; a counter to such misinformation is access to objective information from a trusted health professional.

## Conclusions

The growing epidemic of allergic disease [76] is posing a significant challenge for public health and indicates that a multitude of treatment strategies for allergy will play an increasingly important role in securing population health. Allergen-immunotherapy will undoubtedly comprise a significant component in such efforts, yet promoting this therapeutic intervention will face certain challenges. For one, the time-consuming and inconvenient nature of this therapeutic regimen already leads many patients to abandon treatment prematurely [58]. In this article, it is suggested that additional challenges originating from the growing anti-vaccination movement might also encourage certain patients to oppose allergen-immunotherapy as an appropriate treatment strategy. A reasonable first step in countering this challenge is to prepare allergy specialists for this possibility and provide methods on how to respond to predictable patient fears. Only if clinicians are knowledgeable in vaccines and the anti-vaccination movement will they be prepared to engage in dialogue with an anxious patient and thus, dispel unreasonable associations assumed between allergy treatments and vaccination. This article provides information and guidance to aid clinicians in this situation; however, the global community of allergy specialists should now consider what additional resources, information, and possible collaborations with other health officials (e.g., public health practitioners), will also prove helpful in promoting informed public-perceptions of allergen-immunotherapy. The guidance herein will hopefully serve as the initiator of this needed discussion.

## List of abbreviations

ADRs: adverse drug reactions; GBS: Guillian-Barré Syndrome; IT: allergen-immunotherapy.

## Declaration of competing interests

The authors declare that they have no competing interests.

## Authors' contributions

JB conceived all ideas, conducted all research, and wrote the manuscript.

## Author's Information

JB is a doctoral candidate in Biomedical Sciences specializing in Bioethics, at the University of Montreal. His research interests focus on health policy and public health issues related to the treatment of allergy.
